# Systematic orientation of fresh rectal suction biopsies improves histopathological diagnostics in hirschsprung’s disease – a method description and preliminary report

**DOI:** 10.1186/s12887-023-04048-4

**Published:** 2023-05-17

**Authors:** Emma Fransson, Emilia Gottberg, Rodrigo Munoz Mitev, David Gisselsson, Kristine Hagelsteen, Louise Tofft, Pernilla Stenström, Christina Granéli

**Affiliations:** 1 Department of Pediatric Surgery, Children’s Hospital, Skåne University Hospital Lund, Lund University, Getingevägen 1, Lund, 22185 Sweden; 2 Department of Clinical Genetics and Pathology, Skåne University Hospital, Lund University, Lund, Sweden

**Keywords:** Hirschsprung’s disease, Rectal suction biopsy, Specimen handling, Orienting, Diagnosis

## Abstract

**Background:**

Optimizing rectal suction biopsy (RSB) diagnostics in Hirschsprung’s disease (HD) may shorten diagnostic time and prevent need for repeated biopsies.

**Aim:**

To explore whether systematic orientation of fresh RSB specimens increased biopsy quality, diagnostic times, diagnostic efficacy, and histopathologic workload, and to explore these outcome measures for aganglionic specimens.

**Materials/Methods:**

This was an observational case-control study conducted at a national referral center for HD on data collected from the local HD-diagnostic register. From 2019 each fresh RSB was oriented by the collector in a notch in a foam cushion, placed in a separate cassette, and sent in formalin for pathological analysis. Outcome measures of oriented RSB samples collected 2019–2021 were compared to those of non-oriented RSB samples collected 2015–2018. Staining/immunohistochemistry consisted of hematoxylin eosin, S-100 and calretinin.

**Results:**

78 children with 81 RSBs and 242 biopsy analyzes were included. The frequency of high-quality RSB specimens was higher in oriented: 40% (42/106) versus non-oriented 25% (34/136) (p = 0.018), the diagnostic turnaround time was shorter: 2 days (1–5) versus 3 days (2–8) (p = 0.015), and the number of additional sectioning/leveling/re-orientation per biopsy was lower: 7 (3–26) versus 16 (7–72) (p = 0.011). Specifically for aganglionic specimens, the frequency of high-quality biopsies was generally higher in oriented than in non-oriented RSB specimens: 47% (28/59) versus 14% (7/50) (p < 0.001); the diagnostic efficacy was higher 95% (19/20) versus 60% (9/15) (p = 0.027) and the diagnostic turnaround time shorter: 2 days (2–3) versus 3 days (2–8) (p = 0.036).

**Conclusions:**

Systematic orientation of fresh RSB specimens improves HD diagnostics. Improvement was consistent in aganglionic specimens.

## Introduction

Hirschsprung’s disease (HD) is a congenital disorder with a reported prevalence of approximately 1 in 5000 live births [1]. HD diagnostics rely on histopathological and immunohistochemical analyses, aiming to determine the presence or absence of ganglion cells in the intestinal wall’s submucosa and interstitial myenteric layer. In rectal suction biopsy (RSB) diagnostics, the yielded specimen needs to be deep enough, i.e. including the submucosa, for high-quality representativeness which is required for accurate analysis [2]. However, a recognized obstacle to this is that RSB specimens might be too superficial, i.e. not including the submucosa, for diagnostic efficacy, in which case a repeat biopsy may be necessary [3]. A lower rate of submucosal representation and, therefore, a lower diagnostic efficacy, has been attributed specifically to aganglionic tissue which has been suggested to be less compliant as a result of fibrosis [4]. Other reported factors influencing RSB diagnostic efficacy are the age and weight of the child, and the biopsy collector’s and/or pathologist’s experience, in addition to the RSB-specimen handling with respect to careless treatment of fresh- and formalin-treated specimens, and/or suboptimal/erroneous orientation in histopathologic leveling [3–10]. Any impact of orienting rectal biopsies or RSB specimens on the diagnostic efficacy is, as yet, unknown, but in 1970, an accurate orientation of fresh RSB specimens was suggested to lead to more accurate histopathological analyses [11]. Hypothetically careful treatment and orientation of the small fresh RSB specimen could improve the chance of visualizing the submucosa in RSB tissue samples and thereby increase the diagnostic efficacy. This, in turn, could theoretically potentially reduce diagnostic times or need for re-do biopsies, thus preventing additional suffering for the child and family. Therefore, constituting a part of a collaborative HD-diagnostic improvement project, an orientation method of fresh RSB specimens was developed and introduced in the clinic. The primary aim of the study was to describe this in-house designed method to orient and handle fresh RSB specimens immediately after collection, and to explore whether the systematic orientation increased the biopsy tissue quality and diagnostic efficacy and its effect on diagnostic times and histopathological workload. The secondary aim was to explore whether the outcome improved specifically for aganglionic biopsies.

## Methods

### Patients and data

This was an observational case-control study evaluating an in-house designed method of orienting fresh RSB specimens, and comparing the outcome of oriented versus non-oriented RSB specimens. The study was conducted at a national referral center for HD, serving 5 million inhabitants. Outcomes with regard to diagnostic efficacy, need for re-do biopsy, diagnostic times and histopathological workload were compared between non-oriented and oriented RSB specimens. Non-oriented RSB specimens were collected during the period of January 2015-January 2019 and oriented RSB specimens were collected from February 2019, when the oriented method was implemented, until January 2022. Information about gender, age and child’s weight at the time of biopsy, number of biopsies, need for additional biopsies and number of days from RSB to pathological anatomical diagnosis (PAD) was collected from the Department of Pediatric Surgery’s local diagnostic register. These data included all children undergoing examinations for suspected HD. Information regarding biopsy leveling and re-coloring was collected retrospectively from the pathology department’s charts.

### Biopsy technique and orienting

**Fig. 1:**
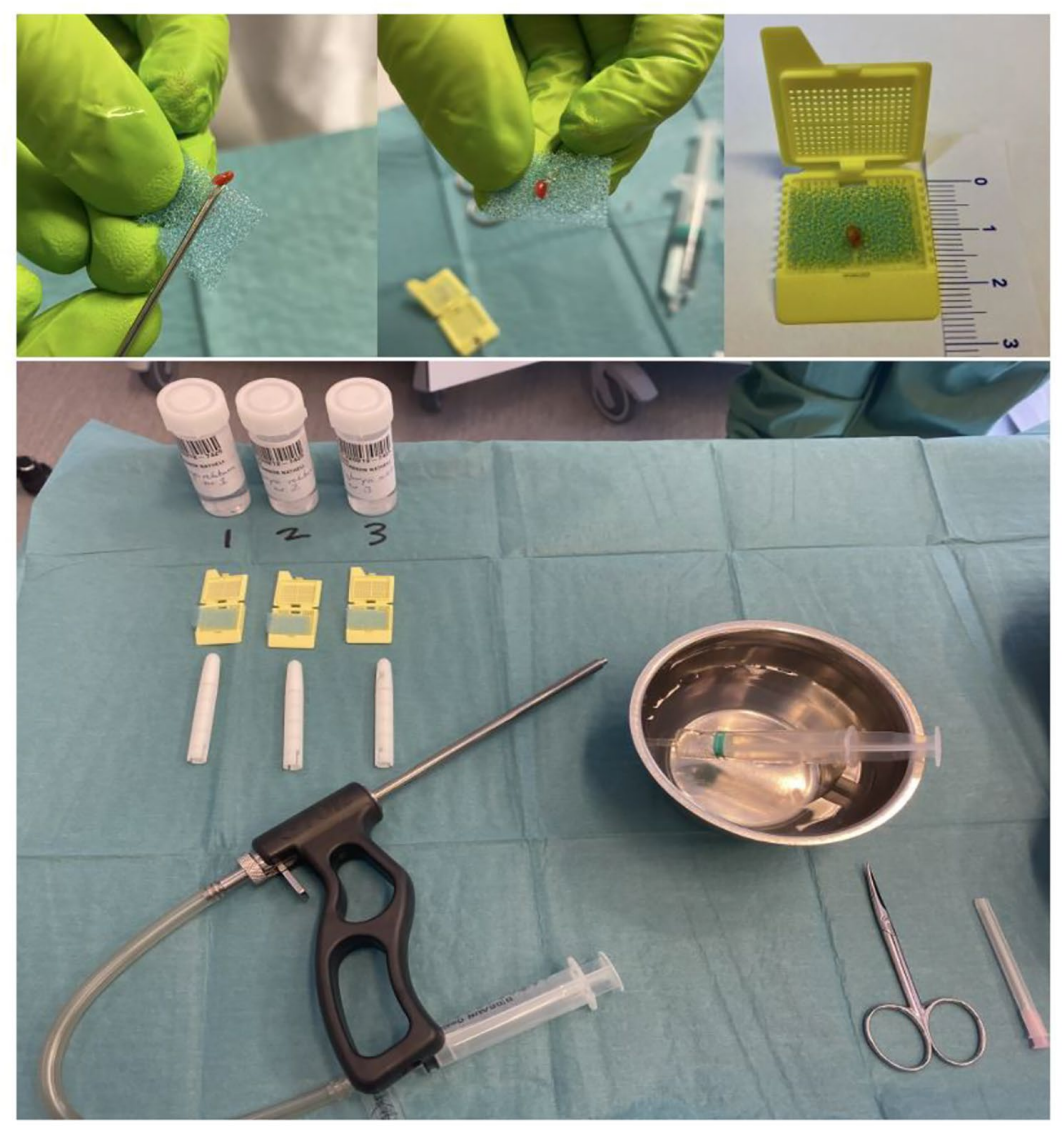
Rectal suction biopsy (RSB) orientation method: Each small RSB specimen is placed carefully within a precut slide in the foam cushion, with its mucosal side in one direction, and its submucosal side in the other. Each foam-cushion pillow is placed within a box, and each box is placed in its own container, pre-filled with formalin, pictured on the upper row. The lower row shows the whole setting for the RSB procedure and orientation

Indications for RSB were symptoms of HD (failure to pass meconium within 48 h, neonatal ileus, vomiting, failure to thrive) and, in most cases, radiology results indicating distal bowel obstruction. RSBs were obtained using a Rbi2→ suction instrument (Aus Systems, Australia) at the outpatient clinic without use of anesthesia or antibiotics. The instrument was calibrated to a negative vacuum of 300 mm H_2_O. Routinely three suction biopsies were taken from the posterior rectal wall, at the levels of 1, 2 and 3 cm, respectively, above the dentate line. On collection, a negative suction vacuum of 200–250 mmH_2_O was used. After collection of the biopsy, the RSB capsule was snapped, and the biopsy removed from the capsule by flushing with saline using a 10 ml syringe. If the specimen was stuck in the capsule, it was extracted carefully with the aid of a BD Microlance 3 needle. From this point, the procedure differed between non-orientation and orientation. Before the orientation procedure was introduced (2019), all three RSB specimens were placed without orientation in the same container prefilled with 10% neutral buffered formalin and sent to the Department of Pathology. From February 2019, fresh RSB specimens were oriented according to the following standard (Fig. 1): immediately after biopsy collection the same pediatric surgeon who took the RSB specimen oriented and positioned the biopsies one by one in a notch in a foam cushion. The biopsy was positioned with the mucosa carefully oriented in one direction (left or right) and the submucosa in the other (Fig. 2a and b) with the help of a needle (BD Microlance 3). Loupe binoculars were used by the surgeon to orient the specimen correctly. The purpose of the notch in the foam cushion was to secure the orientation of the biopsy, as well as to avoid flattening when closing the cassette. The foam cushion was then placed in a separate cassette which, in turn, was placed in a container prefilled with 10% neutral buffered formalin. Each cassette was marked with the level at which the specimen had been taken, recorded as centimeters (1, 2, 3) above the dentate line in the posterior rectal wall regardless of the child’s weight. The information about the biopsy level was sent to the pathologist. Over the study period, seven pediatric surgeons performed RSBs, and four of them oriented RSB specimens.

**Fig. 2:**
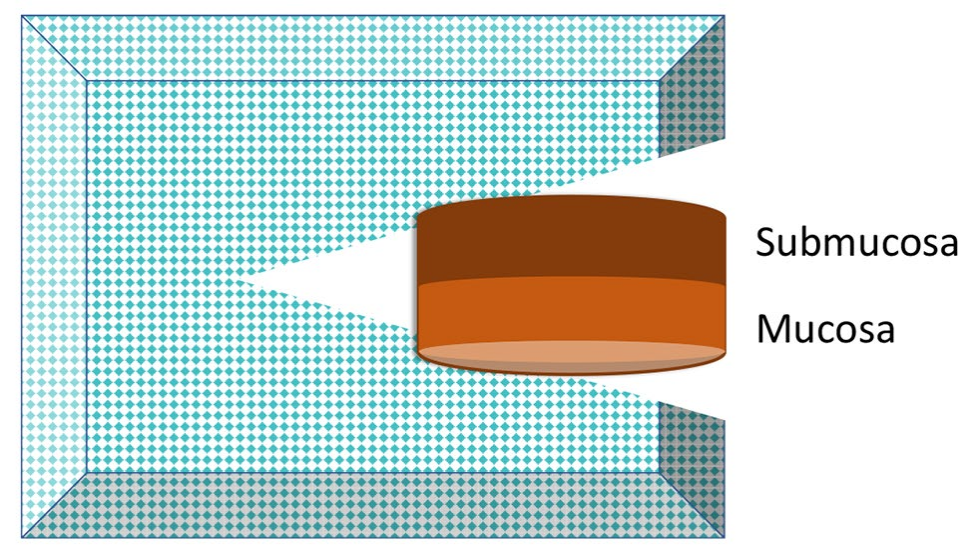
a. Schematic illustration of the rectal suction biopsy (RSB) within the precut slide in the foam cushion, as seen from above

**Fig. 2:**
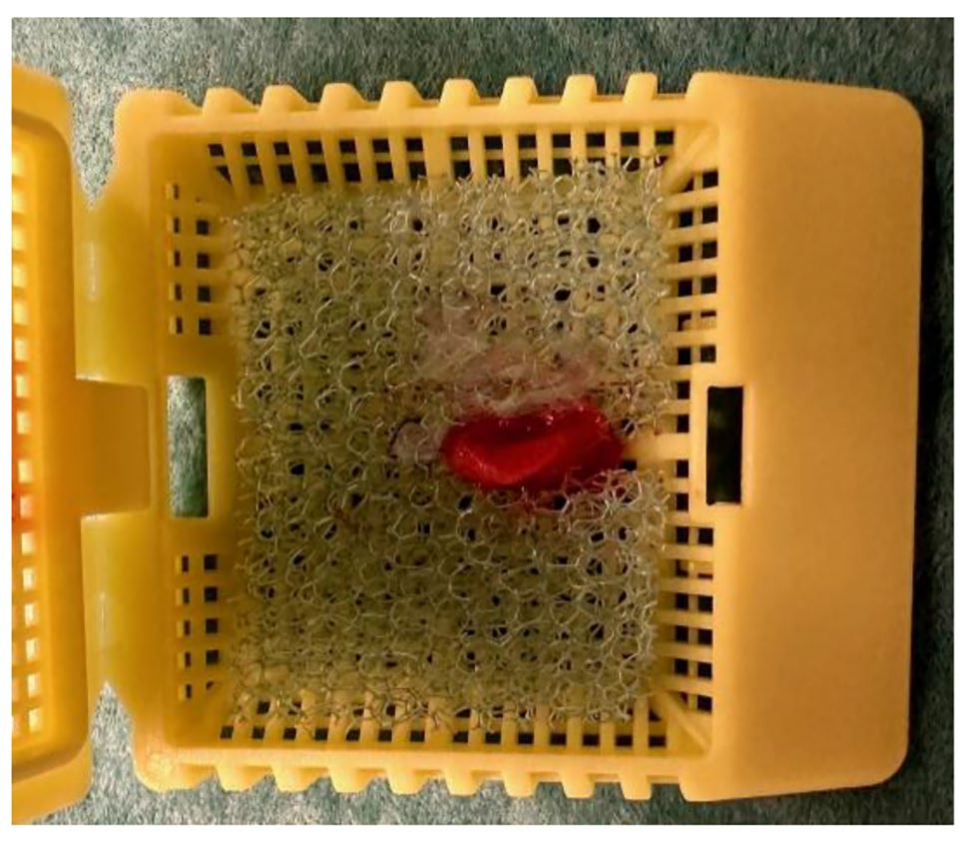
b. Detailed image of the fresh rectal suction biopsy (RSB) specimen placed in the precut slide in the foam cushion

### Biopsy staining and sectioning

**Fig. 3:**
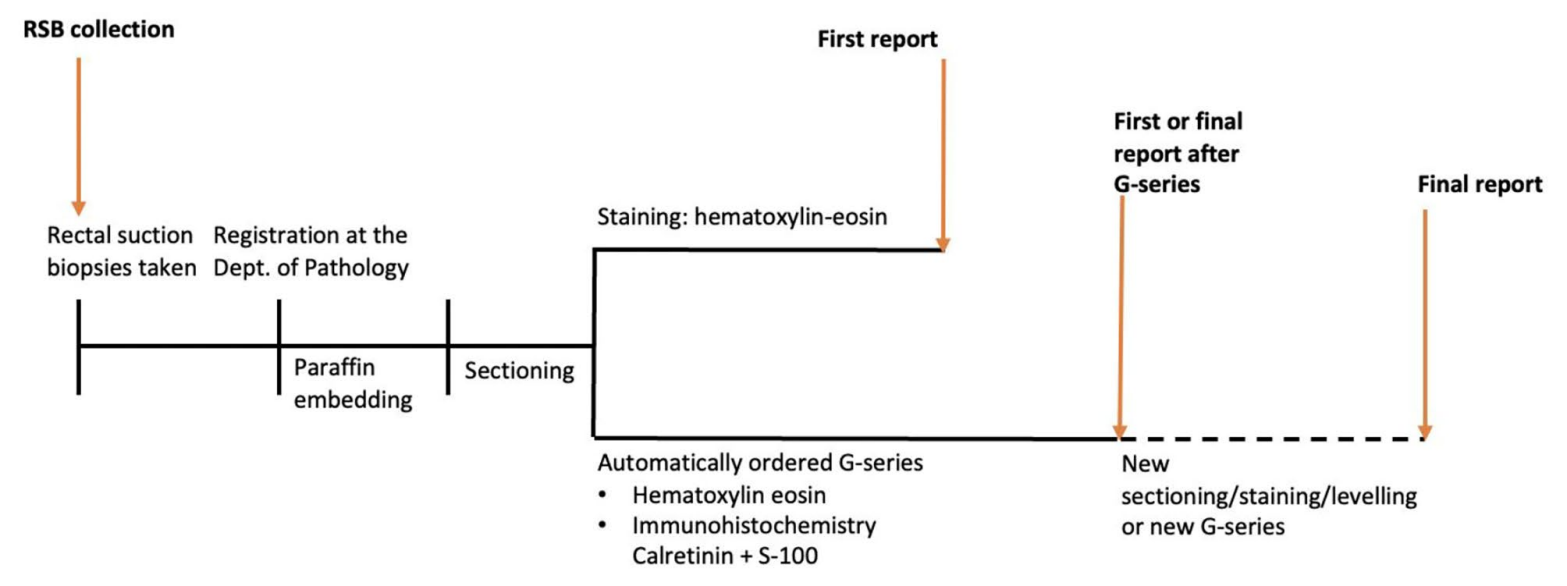
The rectal suction biopsy’s progress from collection to first and final answers at the department of pathology. RSB = Rectal suction biopsy. G-series = ganglion cell series

RSB specimens arriving at the pathology department were routinely embedded in paraffin and thereafter serially sectioned onto microscopy slides. The slides were stained with hematoxylin eosin (HE) and then an automatically ordered staining set: a ganglion cell series (G-series), including HE and immunohistochemistry with S-100 and calretinin was performed. According to the standard protocol the number of HE levels, before the automatically ordered G-series, were two. The HE-stained sections were evaluated for the presence or absence of ganglion cells in the submucosa, and findings were confirmed with G-series to aid in the identification of ganglion cells and nerve fibres in the submucosa, muscularis mucosae or in the lamina propria (Fig. 3). Using standard, automated immunohistochemical methods, S-100 protein was detected by a ready-to-use polyclonal antibody (Ventana, catalog no. 760–2523) and calretinin by the ready-to use monoclonal antibody SP65 (Ventana, catalog no. 790–4467). During the study period, seven pathologists performed all analyzes and reports and two of them, both specialized pediatric pathologists, were responsible for all analyzes and reports.

### Definitions

The outcome of biopsy analyzes were categorized as either being positive for HD, i.e. there was an absence of ganglion cells in an adequate sample and nerve hypertrophia was present, or negative for HD, i.e. ganglion cells were present, and there was no nerve hypertrophia, or inconclusive, i.e. it was not possible to determine the presence or absence of ganglion cells. For the diagnosis of aganglionosis, the following criteria needed to be fulfilled: Absence of ganglion cells, and the presence of S-100 positive nerve fibers that were calretinin negative at the level of the muscularis mucosa. Diagnostic efficacy was defined and assessed as frequency (%) of conclusive RSB specimens. Tissue quality was assessed by a specialist in pathology who reviewed all biopsies after the automatically ordered G-series. Tissue quality was categorized as either high quality or acceptable/low quality tissue. High quality was defined as including the mucosa, muscularis mucosa and submucosa, and not being cross-sectioned in the mucosal plane. Acceptable quality was defined as including a sparse submucosa and/or being cross-sectioned, and being assessable regarding ganglion cells. Low quality was defined as including only mucosa or lymphoid follicles, without submucosa, being cross-sectioned but not assessable regarding ganglion cells. The pathology answers were for the purpose of the study dichotomized as a first or a final answer. A first answer could be given upon the pathologist’s choice along the diagnostic route, before the G-series was complete if the pathologist was sure about the presence or absence of ganglion cells. A first answer was thus defined as an answer, although more examinations were planned and could be given after: (1) HE staining (with planned G-series), or (2) after complete G-series with further examinations planned (reorientation with sectioning and/or leveling). A final answer included (1) single answers after the mandatory HE staining and complete G-series (with no further examinations performed or planned), or (2) confirming a first answer, after the planned further complete G-series or further leveling after such. The histopathological workload was defined as extra work in terms of reorientation and/or deeper leveling, additional to standard HE- and G-series.

### Statistical analysis

All statistical analyzes were performed using SPSS version 28 (IBM Corp, Armonk, New York). Descriptive measurements used were median (range), number (n) and percentage (%). Fisher’s exact test was used for dichotomous categorical data and the Mann-Whitney U-test was used for continuous non-parametric data. A p-value of less than 0.05 was considered to be significant.

## Ethical considerations

The regional research committee approved the study (registration number 2017/191). All methods were carried out in accordance with the relevant guidelines and regulations. Informed consent was collected from all patients’ guardians. With regard to participating in the diagnostic registry, patients’ guardians were informed about their rights to deny registration.

## Results

### Patients and rectal suction biopsies

A total of 82 children examined with RSB for suspected HD were identified in the register. Four children were excluded as a result of having necrotic biopsies (n = 1), cardiofaciocutaneous syndrome (n = 1), infection with intestinal inflammation (n = 1) and RSB taken under anesthesia (establishing stoma) (n = 1). Three children underwent repeated RSBs. Thus, a total of 78 children and a total of 81 biopsy occasions were included. Of these children, 36 (46%) had oriented biopsies and 42 (54%) had non-oriented biopsies corresponding to 37 (46%) and 44 (54%) biopsy occasions, respectively. Overall, 242 biopsies were analyzed and the median number per biopsy occasion was three in both oriented and not-oriented RSBs (Table 1).

**Table 1:** Background information on 78 children with suspected Hirschsprung’s disease (HD) examined on a total of 81 occasions with rectal suction biopsy (RSB), for comparison of outcome. Diagnostic efficacy was defined as % of RSB specimens yielding a conclusive assessment without need for any repeated RSB. Median (min-max), n (%)

	Oriented	Non-oriented	p-value
**Patients (n = 78)**	36	42	
**Gender (girls)**	7/36 (19)	14/42 (33)	
**Biopsy occasions (n = 81)**	37	44	
**Biopsies analyzed per RSB occasion**	3 (1–4)	3 (1–5)	0.569^1^
**Weight at biopsy (kilo)**	3.8 (1.8-8.0)	4.0 (2.4–10.4)	0.306^1^
**Age at biopsy (days)**	26 (2-235)	64 (2-854)	0.202^1^
**Diagnostic efficacy**	36/37 (97)	38/44 (86)	0.119^2^
**Children with aganglionosis**	19/36 (53)	13/42 (31)	0.066^2^

^1^ Mann-Whitney U-test, two-tailed. ^2^ Fisher’s exact test, two-sided

### Diagnostic efficacy

In the oriented biopsy group, one child (1/36) had an inconclusive RSB and a second RSB was performed. In the non-oriented RSB group, six children (6/42) had inconclusive RSBs: two underwent a second RSB while four had a full thickness biopsy, one of them after two repeated RSBs. The indication for re-biopsy was, in all cases, too sparse or absent submucosa. The decision as to whether to perform a full thickness biopsy or not was made by the surgeon. The overall diagnostic efficacy of RSB did not differ statistically between the oriented and non-oriented biopsy group: 97% (36/37) versus 86% (38/44) (p = 0.119).

### Diagnostic times

The frequency of biopsy occasions given a first answer after HE or complete G-series differed significantly between the oriented (20/37) and non-oriented biopsy groups (9/44) (p = 0.002) (Table 2). In the oriented group these first answers (n = 20) were given after HE-staining (n = 16) and after HE and G-series (n = 4), respectively. In the non-oriented biopsy group these first answers (n = 9) were all given after HE-staining. The diagnostic time from the biopsy collection occasion to the first answer was a median of one working day shorter for the oriented biopsies compared to the non-oriented ones (p = 0.015). Similarly, the time from collection to final answer was one day shorter for oriented biopsies (p = 0.002), (Table 2). No incorrect answers on aganglionosis or ganglionosis were reported in the whole cohort, taking into consideration the fact that all first answers turned out to be correct, i.e. confirmed by the full routine investigation, and that no child with ganglionosis was found to have clinically suspected HD at follow-up 1 year after examination.

**Table 2:** Comparison of outcome between oriented and non-oriented fresh rectal suction biopsy (RSB) specimens per biopsy occasion. Diagnostic efficacy was defined as % of RSB occasions yielding a conclusive assessment without need for any repeated RSB. First answer was defined as when the pathologist could answer whether ganglion cells were present or absent, before finishing the G-series (HE, calretinin and S-100). Final answer was defined as answer after full staining routine with HE and automatically ordered G-series. n (%), median (min-max)

	Oriented RSB occasions n = 37	Non-oriented RSB occasions n = 44	p-value
**Diagnostic efficacy n (%)**	36/37 (97)	38/44 (86)	0.119^1^
**First answer n (%)**	20/37 (54)	9/44 (20)	0.002^1^
**Time from biopsy to first answer (working days)**	2 (1–5) n = 20	3 (2–8) n = 9	0.015^2^
**Time from biopsy to final answer (working days)**	4 (2–8) n = 37	5 (2–14) n = 44	0.002^2^
**High-quality RSB specimen/total number of specimens**	42/106 (40)	34/136 (25)	0.018^1^
**Number of biopsy occasions with deeper leveling ordered after the automatically ordered G-series**	14/37 (38)	9/44 (20)	0.137^1^
**Number of levels ordered after the automatically ordered G-series n (range)**	8 (3–26)	16 (7–72)	0.012^2^

^1^ Fisher’s exact test, two-sided. ^2^ Mann-Whitney U-test, two-tailed

### Histopathological workload

The total number of RSB specimens analyzed was 242, distributed as 106 oriented and 136 non-oriented biopsies. The frequency of high-quality RSB specimens was higher in the oriented biopsy group 40% (42/106) compared to the non-oriented group 25% (34/136) (p = 0.018) (Table 2). The number of biopsies in need of additional leveling after the automatically ordered G-series was fewer for oriented biopsies (median 8) than non-oriented ones (median 16) (p = 0.012), (Table 2).

### Evaluation of aganglionic biopsies

In the whole cohort, the overall frequency of children with aganglionosis was 41% (32/78), distributed as 53% (19/36) in the oriented biopsy group and 31% (13/42) in the non-oriented group. In the oriented biopsy group, all children with aganglionosis 19/19 (100%) were diagnosed by RSB. In the non-oriented group, 9/13 were diagnosed by RSB, and 4/9 by full thickness biopsy performed after repeated RSB procedures. In summary, aganglionosis was diagnosed more easily in each patient in the oriented biopsy group: 100% (19/19) compared to 69% (9/13) in the non-oriented biopsy group (p = 0.020).

Regarding diagnostic efficacy, in the oriented RSB group one patient with aganglionosis required a second RSB, corresponding to 20 RSBs. In the non-oriented RSB group, including 13 children, two children had a second RSB corresponding to 15 RSBs. Both re-do RSBs were inconclusive. Thus the diagnostic efficacy per biopsy occasion was higher in the oriented group 95% (19/20) compared to 60% (9/15) in the non-oriented biopsy group (p = 0.027), (Table 3). The aganglionic results were all confirmed by histopathological examination of the resected bowels, and no child presented with HD symptoms or bowel dilation at clinical follow-ups, median 3.4 years (1–7).

**Table 3:** Comparison between oriented and non-oriented rectal suction biopsy (RSB) occasions analyzed for patients with aganglionosis. Overall, 32 children had aganglionosis: 19 in the oriented biopsy group and 13 in the non-oriented biopsy group. Diagnostic efficacy was defined as % of RSB occasions yielding a conclusive assessment without need for any repeated RSB occasion. First answer was defined as when the pathologist could answer whether ganglion cells were present or absent, before the full staining routine was completed. Final answer was defined as the answer after full staining routine with HE and automatically ordered G-series (HE, S100 and calretinin) with or without additional leveling performed. n (%), median (min-max)

	Aganglionic occasions with oriented biopsies (n = 20)	Aganglionic occasions with non-oriented biopsies (n = 15)	p-value
**Diagnostic efficacy**	19/20 (95)	9/15 (60)	0.027^1^
**First answer**	11/20 (55)	5/15 (33)	0.306^1^
**Time from biopsy to first answer** **(working days) **	2 (2–3) n = 11	3 (2–8) n = 5	0.027^2^
**Time from biopsy to final answer (working days)**	4 (2–8) n = 20	5 (2–14) n = 15	0.169^2^
**High-quality specimens/total number of specimens**	28/59 (47)	7/50 (14)	<0.001^1^
**Number of biopsy occasions with deeper levels ordered after the automatically ordered G-series**	7/20 (35)	5/15 (33)	1^1^
**Number of levels ordered after the automatically ordered G-series**	9 (3–26)	22 (11–44)	0.030^2^

For aganglionic RSB, the median time from biopsy collection to the first answer was shorter in oriented (2 days) versus non-oriented (3 days) biopsies (p = 0.027), while the time to reach the final answer did not attain statistical significance (Table 3). Significantly more oriented aganglionic RSB specimens compared to non-oriented ones were of high quality: 47% (28/59) versus 14% (7/50) (p < 0.001), (Table 3). Similarly, the number of additional levels required after the automatically ordered G-series were significantly fewer in the oriented RSB group versus in the non-oriented RSB group; 9 (3–26) versus 22 (11–44), (p = 0.030) (Table 3).

## Discussion

In this study, an in-house designed method of orienting RSB specimens was described and evaluated in the clinic after its implementation. The change of practice in handling RSB specimens, i.e. going from no orientation to a systematic and meticulous orientation of fresh RSB specimens, increased the frequency of high-quality RSB specimens reaching the pathologist, as well as the diagnostic efficacy of such specimens in patients with aganglionosis. Diagnostic times and the need for additional leveling of RSB specimens decreased following the implementation of our technique. To the best of our knowledge, methods of orienting RSB specimens in HD diagnostics, and possible diagnostic effects, have not been described previously.

RSB has the apparent advantage of being able to be performed without anesthesia or discomfort for the child [3, 12]. However, concerns have been raised as lack of submucosa in the microscope after collection, leveling and staining, is reported frequently [3–5]. Reasons for this could be that the biopsy does not include enough of the submucosa, something that has been attributed especially to aganglionic biopsies, age or weight of the child at the time of the biopsy [4, 6–10]. In theory, other reasons for the lack of submucosa in RSB specimens could be that the collected submucosa is partly disturbed after collection, such as due to poor handling, or as a result of the fact that the RSB tissue becomes skewed horizontally when being leveled after paraffin embedding. According to our results, securing a careful handling by orientation led to improved biopsy quality, which was reflected in the shorter diagnostic times and higher diagnostic efficacy, especially for aganglionic RSB specimens. Reduction of diagnostic delay and need for repeated biopsy have been reported to reduce parental anxiety [13], which is of importance in the holistic care of children with HD.

The diagnostic advantage of orienting fresh biopsies correctly immediately after collection is not new information. Within medical areas other than HD, orientation of biopsies has been shown to improve diagnostics: in esophageal examinations of children with gastroesophageal reflux, orientation was shown to improve histological appraisal and increase the diagnostic yield [14] and, in a study on intestinal biopsies, orientation of fresh biopsies was reported to be successful [15]. Transferring this knowledge to RSB, we decided to introduce RSB specimen orientation. This was despite the fact that the overall RSB diagnostic efficacy at our department before implementation of the new technique was 86%, i.e. in line with the literature, which reported RSB efficacies between 70–90% [5, 12, 13, 16–18]. After introduction of the RSB specimen orientation, the overall diagnostic efficacy in our department rose to as much as 97%. Clinically importantly, the increase in efficacy was significant in aganglionic specimens, where the diagnostic efficacy rose from 60 to 95%. This was attributed to the significant rise in high-quality proportion in aganglionic RSB specimens, rising from 14 to 47% in the aganglionic RSB group, as deemed by the pathologist. The findings in the aganglionic group support the theory that handling RSB tissue carefully could be especially important in aganglionic cases where the very limited submucosal content needs to be preserved.

In line with a higher efficacy, shortening of diagnostic times was evident in our study. This could also be attributed to the higher biopsy quality. One of the most important findings was that, if the biopsies were oriented, the pathologists could be more certain of the diagnosis and answer earlier in the process, i.e. after only HE staining. A point that needs to be considered is that diagnostic times could, to a certain extent, also depend on the pathology department’s routines and their priority of HD diagnostics. However, benchmarking our diagnostic times to the literature was not possible since diagnostic times for RSB specimens, to the authors’ best knowledge, have never been presented previously. One reason for the shorter diagnostic times of oriented RSB specimens could be the decreased need for additional leveling and re-orientation. This reduced need for additional biopsy, leveling and re-orientation therefore seemed to lead to an overall reduced workload for the pathologists. In some institutions, orientation of rectal biopsies is performed by the department of pathology, sometimes with the assistance of a microscope. To the authors’ best knowledge, there is no report currently available on such orientation and its implications. Furthermore, it is not known if such orientation should be carried out on a fresh- or formalin-treated specimen. Speculating, the outcome with regard to diagnostic efficacy and reduced workload could be expected to be similar to ours, regardless of where a structured orientation is performed. In addition, positioning of in foam cushion could possibly be a useful diagnostic technique also for frozen biopsy taken during surgery.

A strength of the study is the orientation method’s generalizability, and its easy performance and feasibility. In addition, any selection bias at the department of pathology, such as of biomedical technicians who performed the paraffin embedding and staining, could be excluded since the study was blinded for biotechnicians who therefore just performed their routine work. Another strength was the high and complete covering rate, attained thanks to the local prospectively collected HD-diagnostic register. Also, the same histo-pathological and -immunological staining procedure (HE, S-100 and calretinin) was used during the whole study period.

The main limitations were the relatively small number of patients and the fact that two time periods were compared, instead of randomization. Another weakness was the bias with regard to diagnostic times, where pathologists might have endeavored to provide answers more quickly than before orientation was introduced. This could be of particular concern since a first answer was given upon the pathologist’s choice. In the clinic, the safety of answering before the full G-series is finalized needs to be discussed carefully within each institution’s setting. In the present situation with experienced pediatric pathologists with a close clinical collaboration to the pediatric surgeons, no incorrect answers were given. It is also worth mentioning that this study only included histopathological analyses on specimens stained with HE, S-100 and calretinin, i.e. the hospital’s standard diagnostic techniques. In demonstrating aganglionosis and hypertrophied nerves, i.e. the main features of HD, acetylcholine esterase constitutes an optional staining technique; however, this was not included in this study.

Despite several study limitations, we showed that orienting fresh RSB specimens increased the quality and diagnostic efficacy, in parallel with reducing diagnostic times and the additional histopathological work. Therefore, RSB specimen orientation seems to reduce a stressful waiting time for families, bringing an earlier and optimized treatment for children, especially those with aganglionosis.

## Conclusion

This first report on orienting RSB specimens shows that the in-house designed method of a systematic and meticulous orientation of fresh RSB specimens encompasses several advantages: it increases the frequency of high-quality RSB specimens, reduces the diagnostic time and limits the histopathological workload. Importantly, orientation of RSB specimens can improve the diagnostic efficacy if aganglionosis is present.

## Data Availability

The datasets generated during and/or analyzed during the current study are available from the corresponding author on reasonable request.
